# Laboratory strengthening strategies to advance drug susceptibility testing for BPaL regimens in TB treatment

**DOI:** 10.5588/pha.25.0014

**Published:** 2025-12-03

**Authors:** J-K. Jung, J.S. Lee, A. Slyzkyi, D.F. Wares, S.N. Cho

**Affiliations:** 1 International Tuberculosis Research Center, 236 Gaposunhwan-ro, Masanhappo-gu, Changwon-si, Gyeongsangnam-do, Republic of Korea;; 2 KNCV Tuberculosis Foundation, Postbus 146, 2501, CC Den Haag, The Hague, The Netherlands.

**Keywords:** tuberculosis, drug-resistant tuberculosis, DST, LIFT-TB, The Philippines, Myanmar, Indonesia, Vietnam, Uzbekistan, Kyrgyzstan, Ukraine

## Abstract

**BACKGROUND:**

To support BPaL (bedaquiline [Bdq], pretomanid [Pa] and linezolid [Lzd]) rollout, countries require ongoing technical and policy support for standardized drug susceptibility testing (DST).

**METHODS:**

The Leveraging Innovation for the Faster Treatment of Tuberculosis (LIFT-TB) operational research project aimed to strengthen laboratory capacity for DST in 7 countries (the Philippines, Myanmar, Indonesia, Vietnam, Uzbekistan, Kyrgyzstan and Ukraine) through needs assessments, reagent and equipment support, quality control and training.

**RESULTS:**

During the project, we trained 157 professionals in phenotypic and molecular DST, enhancing quality assurance and implementation. We found there was variable DST capacity and resistance patterns.

**CONCLUSION:**

Our study highlights the need for continued investment in training and infrastructure to integrate DST into routine diagnostics and to support scale-up of BPaL regimens in high TB-burden settings.

TB is one of the top 10 deadliest diseases and in 2023, it replaced COVID-19 and returned to being the leading cause of death from a single infectious agent.^1^ In 2023, the WHO estimated 10.8 million TB cases and 400,000 rifampicin-/multidrug-resistant TB (RR-/MDR-TB) cases.^1^ Discovered in 2000, pretomanid (Pa) was developed as an anti-TB drug by TB Alliance, USA. The Nix-TB trial showed that the 6-month oral BPaL (bedaquiline [Bdq], Pa, and linezolid [Lzd]) regimen had a 90% success rate in 109 extensively drug-resistant TB (XDR-TB; based on the pre-2021 WHO definition) and treatment-intolerant MDR-TB patients.^2^ In 2019, these results led to BPaL approval by the Food and Drug Administration (FDA) in the USA, and the European Medicines Agency in 2020. The 2020 WHO guidelines endorsed its use under operational research (OR), emphasizing drug susceptibility testing (DST) and safety monitoring.^3^ Following WHO's recommendations, the ‘Leveraging Innovation for the Faster Treatment of Tuberculosis’ (LIFT-TB) project was launched across seven countries. Co-funded by the Korean International Cooperation Agency and TB Alliance, this study examines BPaL’s efficacy and safety for XDR-TB and MDR-TB. Within this project, the International Tuberculosis Research Center (ITRC) was responsible for enhancing the diagnostic capacity of laboratories participating in the OR (see Supplementary Data). Here we outline the stepwise approach to laboratory capacity building and its impact on DST capacity, drawing on observations from the OR implementation, surveys, reports and discussions during LIFT-TB laboratory training.

## METHODS

### Guideline adaptation and DST training framework

**FIGURE 1.**
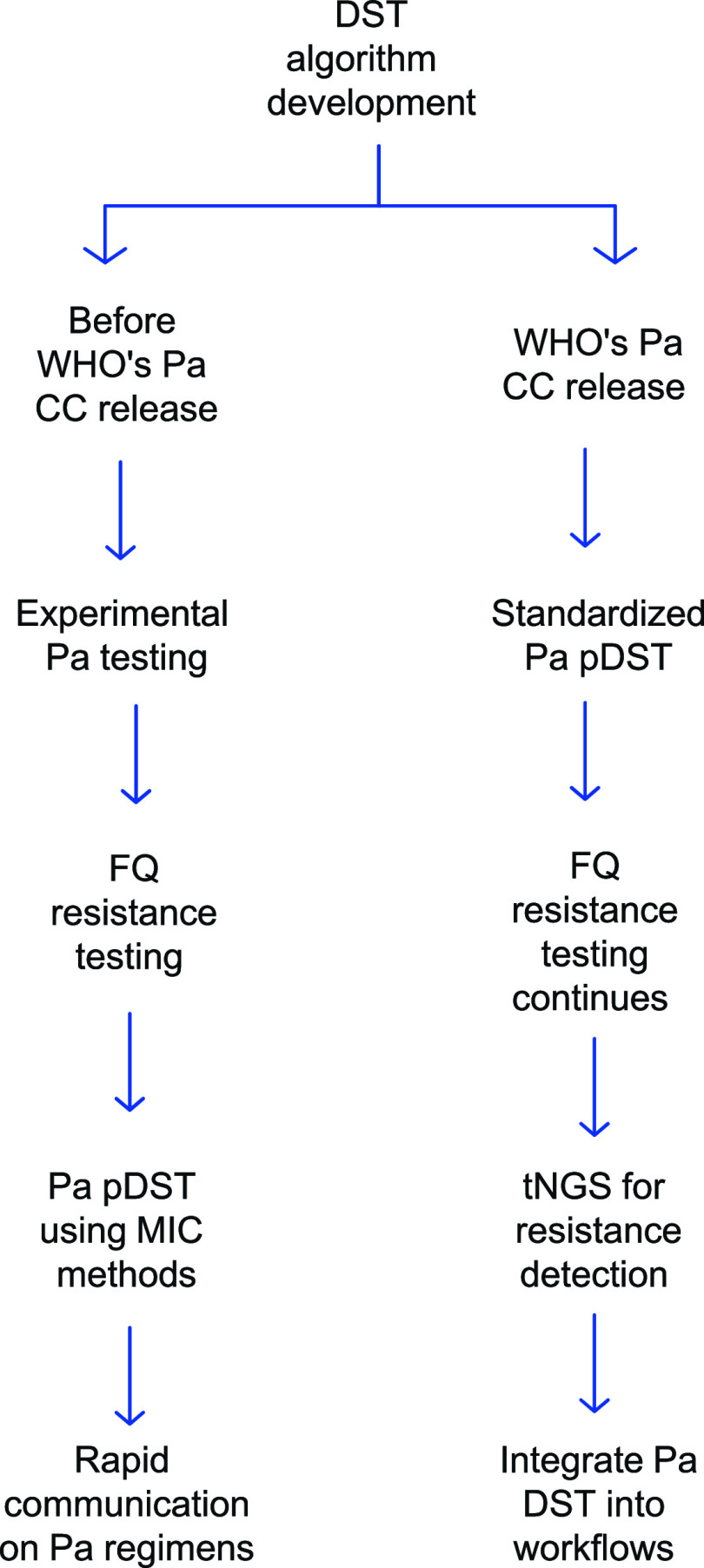
Drug susceptibility testing algorithm for drug-resistant TB (DR-TB).

Training programs were adapted to the DST framework in the WHO guidelines existing at the time, integrating phenotypic and molecular testing to standardize DR-TB diagnostics with evolving recommendations (Figure 1).^3,4^

#### DST before WHO’s Pa critical concentration release

Before the WHO established the critical concentration (CC) values for Pa in December 2023, Pa DST lacked standardization and was primarily used in OR.^4^ Without WHO-approved resistance breakpoints, minimum inhibitory concentration (MIC) based phenotypic DST (pDST) varied in interpretation,^5^ and fluoroquinolone (FQ) resistance testing determined BPaL eligibility, using line probe assay (LPA) for screening and MGIT-based pDST for confirmation.^3^ To compensate for this gap, some laboratories used Pa-resistant *Mycobacterium tuberculosis* (*Mtb*) isolates for quality assurance (QA) and applied molecular DST (LPA, sequencing) to detect potential resistance mutations, although the WHO did not endorse these for treatment decisions.^4^ In 2022, WHO recommended the programmatic use of Pa-containing regimens, but Pa DST was not required for treatment initiation, and FQ resistance testing remained the primary determinant for BPaL eligibility.^4^

#### DST implementation following WHO’s update

WHO’s update in 2023 introduced CC values for Pa, enabling standardized pDST using Mycobacteria Growth Indicator Tube (MGIT) and improving resistance classification.^4^ However, FQ resistance testing remains essential for BPaL eligibility, with LPA for screening and MGIT-based pDST or targeted next-generation sequencing (tNGS) for confirmation.^4^ WHO further promoted tNGS for detecting resistance-associated mutations in Bdq, Lzd and Pa, reflecting a shift toward genotypic testing in settings where pDST remains challenging.^6,7^ WHO also advised National TB Programs (NTPs) to integrate Pa DST into routine DR-TB diagnostics, alongside Bdq, FQ and Lzd DST, to support individualized treatment selection.^4^

### Technical support for laboratory strengthening

**FIGURE 2.**
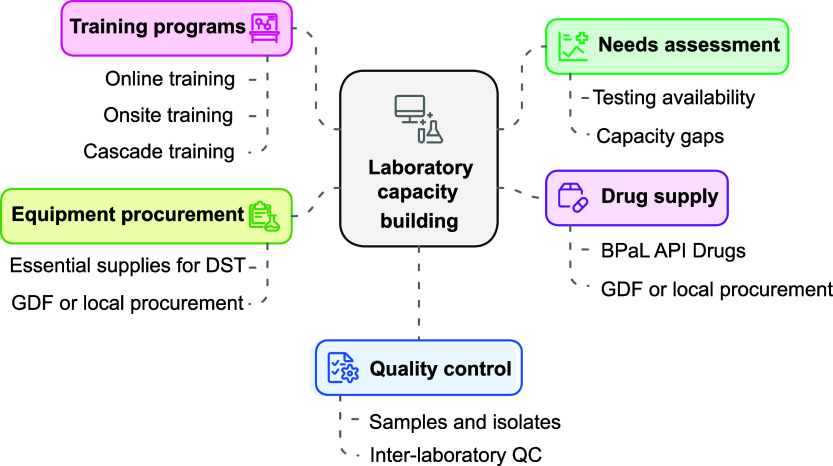
Laboratory strengthening framework.

Throughout the BPaL OR, a key focus was on strengthening DST capacity across participating countries. The laboratory support strategy included needs assessments, equipment and reagent procurement, QA, and a structured training program to ensure the successful implementation of pDST and molecular DST techniques for BPaL drugs with a focus on the new drug, Pa (Figure 2).

#### Laboratory needs assessment

The laboratory needs assessment was conducted in three phases (2020, 2021 and 2022) to evaluate DST capacity gaps and guide laboratory strengthening efforts in the participating countries. The assessment covered six key domains (Supplementary Data). Whilst pDST for BPaL was available in most reference laboratories, it was not accessible in many OR sites, necessitating sample transfers for testing. GeneXpert MTB/RIF (Ultra) was widely available, but GeneXpert XDR and HAIN MTBDRsl were either absent or limited in several countries, restricting the ability to detect FQ and second-line drug resistance. Quality control programs showed significant variability, with external QC largely reliant on international partners, including RIT-Japan, NIRT-India, and Gauting-Germany. While laboratory standard operating procedures (SOPs) and equipment maintenance plans were generally in place, power backup systems remained inconsistent, highlighting the need for improved infrastructure to ensure uninterrupted diagnostic services (Table 1).

**TABLE 1.** Main findings of the laboratory needs assessment.

Category	Findings	Countries affected
**pDST for BPaL**	Available in reference laboratories, but OR sites lacked capacity	Indonesia, Vietnam
**GeneXpert XDR**	Not available in multiple sites	Indonesia, Vietnam, Kyrgyzstan
**HAIN MTBDRsl**	Available in some reference laboratories but absent in most OR sites	Philippines, Indonesia
**External QC**	Dependent on international providers	Philippines (RIT Japan), Myanmar (NIRT India), Vietnam (MRL Australia), Uzbekistan (Gauting Germany)
**SOPs and maintenance**	Power backup not consistently secured	Indonesia

SOP = standard operating procedure

#### Drug and equipment supply

ITRC supplied BPaL drugs twice for laboratory use before on-site training and after the 2022 WHO guideline update. Pa was received from TB Alliance, while Bdq and Lzd were procured through a South Korean supplier. Since 2023, Lzd has been procured via the Global Drug Facility (GDF), except in countries requiring local drug procurement.

In parallel, ITRC procured a wide range of DST equipment and consumables, mainly through GDF, except for Myanmar and Indonesia, where local procurement was required. The requested items included pipettes, centrifuges, laboratory freezers, gene analysis equipment, bacterial culture supplies, and reagents essential for sample processing, incubation, and molecular research.

#### DNA samples and Mtb isolates for external QC

Pa-resistant Mtb isolates were provided to the Philippines, Indonesia, Kyrgyzstan, Ukraine, and Uzbekistan. In collaboration with the Gauting laboratory, ITRC prepared a QA panel of genotypically and phenotypically characterized *Mtb* strains, which was distributed for inter-laboratory QC in Kyrgyzstan, Ukraine, and Uzbekistan. Participating laboratories successfully passed Pa pDST inter-laboratory QC, but at the time, no consensus on Pa CC existed, and thus, official EQA recommendations were unavailable. The Philippines requested internal LPA QC materials due to issues with nonspecific reactions, control failures, and unusual signal patterns. In response, ITRC prepared and provided a specialized QC panel for LPA performance validation.

#### Laboratory training and capacity building

To enhance laboratory proficiency in phenotypic and molecular DST, a hybrid training model was implemented, combining online, onsite, and cascade training approaches. Training materials, including MGIT DST for Pa, were made available in multiple languages (English, Russian, Bahasa and Vietnamese) on the ITRC website (http://www.itrc.kr/en/sub/research/health.asp). Once COVID-19-related travel restrictions were lifted, hands-on pDST training was conducted across the 7 project countries.

## RESULTS

### Training for DST capacity strengthening

A total of 157 laboratory professionals participated in various training sessions, covering pDST for Bdq, Lzd and Pa as well as molecular DST techniques (Table 2). The cascade training approach in the Philippines and Uzbekistan helped further expand pDST capacity beyond central reference laboratories. Following these efforts, all project countries initiated pDST for BPaL drugs, although the level of implementation varied.

**TABLE 2.** Laboratory staff trained.

Country	LPA (remote, 2021-2022)^A^	pDST (onsite, 2022-2023)	tNGS (online, 2023)	pDST (cascade to regional labs, 2024)	pDST (refresher, onsite, 2024)^B^	tNGS (onsite, 2024)^B^
**Indonesia**		9	6		2	2
**Kyrgyzstan**		9	4		2	2
**Myanmar**	11	15	1			
**Philippines**	19	7	18	3	3	3
**Ukraine**		1**^C^**	2		2	2
**Uzbekistan**		10	2	13	2	2
**Vietnam**		12	4			
**Total**	**30**	**63**	**37**	**16**	**11**	**11**

A During COVID-19 travel restrictions, training was given remotely using pre-recorded materials and live Q&A sessions.

B Since pDST refresher and tNGS onsite trainings took place simultaneously, numbers are not double-counted**.**

C Hosted at the Gauting laboratory in Germany.

### Implementation of phenotypic DST for BPaL

Pa MIC data were available only from the Philippines and Uzbekistan during the assessment period (Table 3). In the Philippines, MIC results showed that 38% (18/48) of isolates had MIC ≥2 µg/mL. Uzbekistan reported MIC results for 49 isolates, with 43 (88%) showing MIC ≤0.5 µg/mL. In other countries, Pa DST data were not reported or unavailable, often due to technical or logistical constraints.

**TABLE 3.** pDST results by country.

Country	Bdq DST tests	Lzd DST tests	Pa DST tests	Bdq resistance cases	Lzd resistance cases	Pa MIC results (µg/mL)
**Indonesia**	203	203	203	1 (0.5%)	1 (0.5%)	not reported**^A^**
**Kyrgyzstan**	843	843	843	22 (2.6%)	17 (2.0%)	not reported**^A^**
**Philippines**	48	48	48	11 (22.9%)	0	≤0.5 (16), 0.5–<2 (14), 2 (15), >2 (3)
**Ukraine**	3,417	3,417	not available**^B^**	9 (0.3%)	41 (1.2%)	not available**^B^**
**Uzbekistan**	49	49	49	1 (2.0%)	2 (4.1%)	≤0.5 (43), 0.5–<2 (4), 2 (2)

A ’not reported’ refers to cases where testing was performed but MIC results were not recorded or included in the dataset.

B Ukraine’s Pa DST and Pa Mic results were not available due to logistical and operational barriers that delayed implementation until late 2024.

Between 2022 and early 2024, laboratories performed pDST and molecular DST for BPaL drugs using the MGIT system. In Indonesia, 203 isolates were tested, detecting Bdq and Lzd resistance in one case each (0.5%); Pa MIC results were not reported. Kyrgyzstan conducted Bdq, Lzd, and Pa DST on 843 isolates, detecting 22 Bdq-resistant and 17 Lzd-resistant cases; however, Pa MIC results were not reported. In the Philippines, Bdq resistance was observed in 11 of 48 isolates; Lzd resistance was none. Ukraine reported 3,417 DR-TB cases, detecting 9 Bdq-resistant (0.3%) and 41 Lzd-resistant cases (1.2%); Pa DST was not available. In Uzbekistan, resistance to Bdq (2.0%) and Lzd (4.1%) was detected, with Pa MIC data reported as described above.

### Country-specific trends and key findings

The findings show varied resistance patterns and DST implementation. In the Philippines, a notably high Bdq resistance rate (22.9%) suggests pre-existing resistance before BPaL scale-up. Indonesia’s low resistance rates support current treatment strategies. Uzbekistan’s results indicate feasibility, but further standardization of Pa MIC testing and reporting is needed. Kyrgyzstan’s high Bdq resistance emphasizes the importance of expanding molecular DST. Ukraine’s low Bdq resistance (0.3%) but moderate Lzd resistance (1.2%) calls for continued monitoring.

Despite overall progress, there were limitations to our study and notable data gaps remain. Myanmar and Vietnam did not submit DST results even after receiving technical support, and although Ukraine submitted Bdq and Lzd DST data, Pa DST was unavailable due to an implementation delay. These gaps limit the completeness of regional comparisons.

## CONCLUSION

Hybrid training and tailored laboratory support, based on needs assessments, strengthened DST capacity across the seven project countries. However, Pa DST was not implemented in all settings due to persistent barriers, including infrastructure limitations and service interruptions. Preliminary findings demonstrate expanded use of pDST and molecular DST; however, integration of Pa DST into routine diagnostics remains limited. Continued support is needed for all countries, particularly Myanmar and Ukraine, where laboratory functions were hindered.

To support BPaL rollout, countries require ongoing technical and policy support for standardized Pa DST implementation, aligned with WHO’s recommendations for critical concentrations. Decentralizing DST via subnational cascade training will enhance resistance detection, inform treatment decisions, and support national scale-up of BPaL-based regimens. Sustained investment in training, QA systems, and molecular testing is essential for establishing Pa DST as a standardized component of DR-TB management.

## Supplementary Material


