# Supplementary material for: Laboratory strengthening strategies to advance drug susceptibility testing for BPaL regimens in TB treatment
Source: Public Health Action. 2025 Dec 3;15(4):145–8. doi: 10.5588/pha.25.0014 (PMC12687124; doi:10.5588/pha.25.0014)
Supplement: Supplementary file 1 [file pha25-0014_supplementarydata1.pdf]

Annex 1. Reference Laboratories and OR Sites involved in the LIFT-TB Operational Research

The reference laboratories and OR sites played a critical role in supporting DST implementation and capacity-building efforts for the BPaL regimen introduction. The table below outlines the national reference laboratories and OR sites involved across the seven participating countries.

Country	Reference Laboratory	OR Sites	Number of OR Sites
**Indonesia**	BBLK Surabaya	RSUP, RSPI, RSUD, Laboratorium Mikrobiologi Klinik RSUP	4
**Kyrgyzstan**	National Reference Laboratory of National Center for Phthisiology	Same as the reference laboratory	1
**Myanmar**	National TB Reference Laboratory	Same as the reference laboratory	1
**Philippines**	National TB Reference Laboratory	LCP, DJNRMH, LUMC, R1MC, DDVMH, JBLMRH, CLCHD, BaMC, PTSI, SMMGHSC, WVMC, ECS, ZCMC, DRTL, NMMC, NMTRL, PMP, SPMC	12
**Ukraine** *	Not specified	3 sites (not individually identified)	3
**Uzbekistan**	National Reference Laboratory RSSPMCPhP	Bukhara Regional Culture Laboratory, Fergana Regional Culture Laboratory, Samarkand Regional DST TB Laboratory, Andijan LPA-HAIN Laboratory	4
**Vietnam**	NLH National TB Reference Laboratory	Hanoi Lung Hospital, Pham Ngoc Thack HCMC, Can Tho Hospital	3

** Ukraine's OR sites were identified by number only, without individual site details provided.*

Annex 2. Laboratory Needs Assessment Key Domains

Assessment Area	Key Focus	Specific Aspects Assessed
**DST capacity**	Available diagnostic methods	Rapid molecular DST (second line-LPA, pDST, GeneXpert, bacterial culture, external quality assurance [EQA])
**Quality Assurance**	Internal and external quality control (QC)	QC programs, proficiency testing participation
**Laboratory standard operating procedures (SOPs) and manuals**	Standardization	Availability of SOPs, adherence to WHO guidelines
**Equipment and maintenance**	Infrastructure assessment	Equipment availability, maintenance plans, power backup
**Procurement and supplies**	Stock management	Availability of reagents, Bdq/Lzd/Pa API drugs, and EQA materials
**Training needs**	Capacity building	Need for phenotypic and molecular DST training
